# Redo off-pump coronary artery bypass grafting via a left thoracotomy

**DOI:** 10.5830/CVJA-2014-06

**Published:** 2015

**Authors:** Ibrahim Duvan, Sanser Ates, Burak Emre Onuk, Umit Pinar Sungar, Murat Kurtoglu, Yahya Halidun Karagoz

**Affiliations:** Department of Cardiac Surgery, Guven Hospital, Ankara, Turkey; Department of Cardiac Surgery, Guven Hospital, Ankara, Turkey; Department of Cardiac Surgery, Guven Hospital, Ankara, Turkey; Department of Cardiac Surgery, Guven Hospital, Ankara, Turkey; Department of Cardiac Surgery, Guven Hospital, Ankara, Turkey; Department of Cardiac Surgery, Guven Hospital, Ankara, Turkey

**Keywords:** coronary artery bypass grafting, re-operation, circumflex artery, thoracotomy

## Abstract

**Background:**

In this study, we retrospectively reviewed our experience in a meticulously selected group of patients undergoing redo off-pump coronary artery bypass graft (CABG) surgery from the descending aorta to the circumflex artery (Cx) and its branches.

**Methods:**

Between January 2001 and October 2013, 32 patients at our hospital underwent redo off-pump CABG from the descending aorta to the Cx and its branches via a left posterolateral thoracotomy. Of these patients, 27 were male (84.3%) and five were female (15.7%), with a mean age of 61.66 ± 8.63 years. All patients had a patent left internal thoracic artery-to-left anterior descending coronary artery (LITA–LAD) anastomosis. Thoracotomy was performed through the fifth intercostal space. The saphenous vein or radial artery was prepared as a graft at the same time as the left posterolateral thoracotomy from the contralateral extremity, without any positional problem.

**Results:**

The main reasons for surgery in this group of patients were new lesion formation in 19, graft occlusion in six, and both in seven patients. The average operating time was 143.90 ± 36.93 minutes, respiratory assist time was 5.08 ± 1.88 hours, intensive care unit (ICU) stay was 21.3 ± 4.41 hours and hospital stay was 5.06 ± 2.74 days. Thirty-eight bypasses were performed. The follow-up period was 56.17 ± 39.2 months. Six patients were lost in the follow-up period and four patients died. Twenty-two were alive and free of cardiac problems.

**Conclusion:**

Redo off-pump CABG via a left posterolateral thoracotomy provided a safe and effective surgical approach with lower rates of postoperative morbidity and mortality in patients who required revascularisation of the Cx and its branches.

## Abstract

Re-operative coronary artery bypass graft (CABG) surgery is more complicated than the initial CABG and it may also be more hazardous because of risk factors related to median resternotomy, such as cardiac injury and damage to the patent grafts due to sternal adhesion.^1^ Deciding on the appropriate treatment for recurrent coronary artery disease (CAD), especially conditions such as non-left anterior descending coronary artery (LAD) ischaemic lesions during the existence of patent left internal thoracic artery-to-left anterior descending coronary artery (LITA–LAD) anastomosis is a dilemma.^2^

If the patient is unresponsive to medical therapy, and percutaneous transluminal coronary angioplasty (PTCA) and/or stenting is not appropriate for revascularisation, alternative surgical strategies, excluding resternotomy and cardiopulmonary bypass (CPB), may be the most appropriate way of revascularising the branches of the circumflex artery (Cx) or right coronary arteries (RCA) (non-LAD territories).^3^-^5^ In selected patients, off-pump redo CABG for the branches of the Cx via a posterolateral thoracotomy may reduce the risks due to median resternotomy and dissection of the heart.

This procedure to avoid resternotomy and CPB has become an established and popular way of revascularising recurrent coronary artery disease in the lateral aspect of the heart. In this article, we share our experience of 32 patients who underwent redo CABG for the Cx and its branches via a left posterolateral thoracotomy.

## Methods

Between January 2001 and October 2013, 32 off-pump CABG re-operations via a posterolateral thoracotomy for the branches of the Cx system were performed at the Department of Cardiac Surgery of Guven Hospital in Ankara, Turkey. During this time, 450 patients underwent isolated redo off-pump CABG and our study group constituted 7.1% of this population. There were 27 men and five women, aged 61.66 ± 8.63 years, with a mean of 40–76 years (Table 1).

**Table 1:** Pre-operative demographic data

*Variables*	*Demographic data (n = 32)*
Age (years) (mean)	61.66 ± 8.63 (40–76)
Male, n (%)	27 (84.4)
Female, n (%)	5 (15.6)
Hypertension, n (%)	21 (65.6)
Smoking, n (%)	21 (65.6)
Diabetes mellitus, n (%)	13 (40.6)
Family history, n (%)	22 (68.7)
Hyperlipidaemia, n (%)	22 (68.7)
Myocardial infarction, n (%)	11 (34.3)
COPD, n (%)	6 (18.7)
CVA, n (%)	2 (6.2)

COPD, chronic obstructive pulmonary disease; CVA, cerebrovascular accident.

Co-morbidity factors of the patients were pre-operative hyperlipidaemia, family history, smoking, hypertension, diabetes mellitus, chronic obstructive pulmonary disease and cerebrovascular disease. The period between the first and redo operation via thoracotomy was 103.03 ± 63.33 months (20–264). Only one patient was operated on three times, the others were operated on twice. There were 2.16 ± 1.019 anastomoses performed in each of the previous operations and the total number of anastomoses was 67, whereas the number of patent anastomoses was 44. All of the LITA-to-LAD anastomoses were patent and 10 of the RCA and two of the Cx system anastomoses were also patent.

**Fig. 1.**
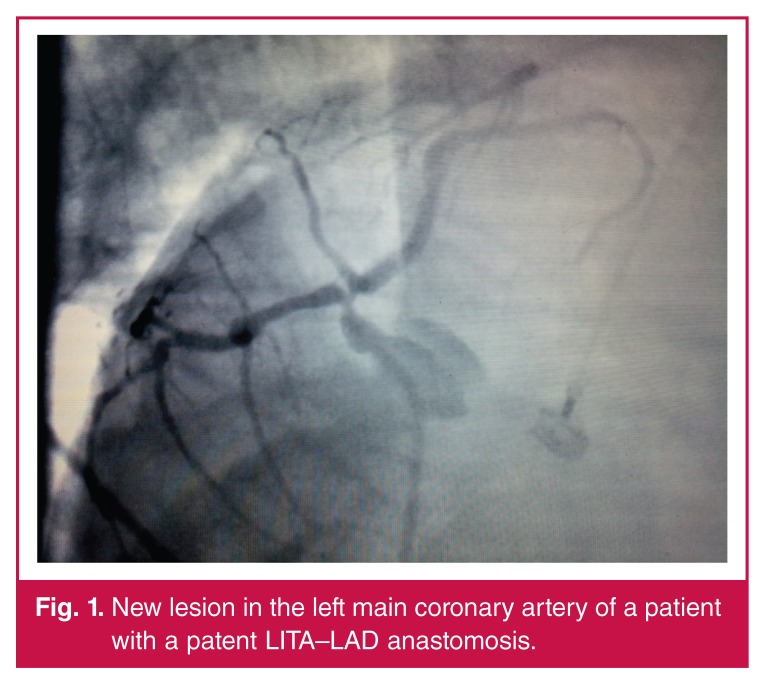
New lesion in the left main coronary artery of a patient with a patent LITA–LAD anastomosis.

Patients had symptoms of angina, depending on a problem in the Cx system, and unfortunately medical therapy was unsuccessful. Six had already been revascularised by both PTCA and stent. PTCA only was performed in four of the patients. Complete revascularisation is the first priority universally for all patients in cardiac surgery, so these were all candidates for redo CABG. The reason for ischaemic symptoms in six of our patients was graft occlusion, new lesions in 19, and both in seven (Table 2). New lesions occurred in the left main coronary arteries of eight patients (Fig. 1), and in the rest, in branches of the Cx.

**Table 2:** Information on the patients after the first operation up to the redo Cx CABG via thoracotomy

*Variable*	*Number ± SD*
Number of previous grafts	2.1667 ± 1.019
Patent anastomoses	
LIMA–LAD	32
RCA	10
Cx	2
Reason of redo Cx CABG	
Graft occlusion	6
New lesion	19
Both	7
Interventions	
PTCA	4
PTCA + stent	6
Period between the first and redo CABG via thoracotomy operation (months)	103.03 ± 63.33

**Fig. 2.**
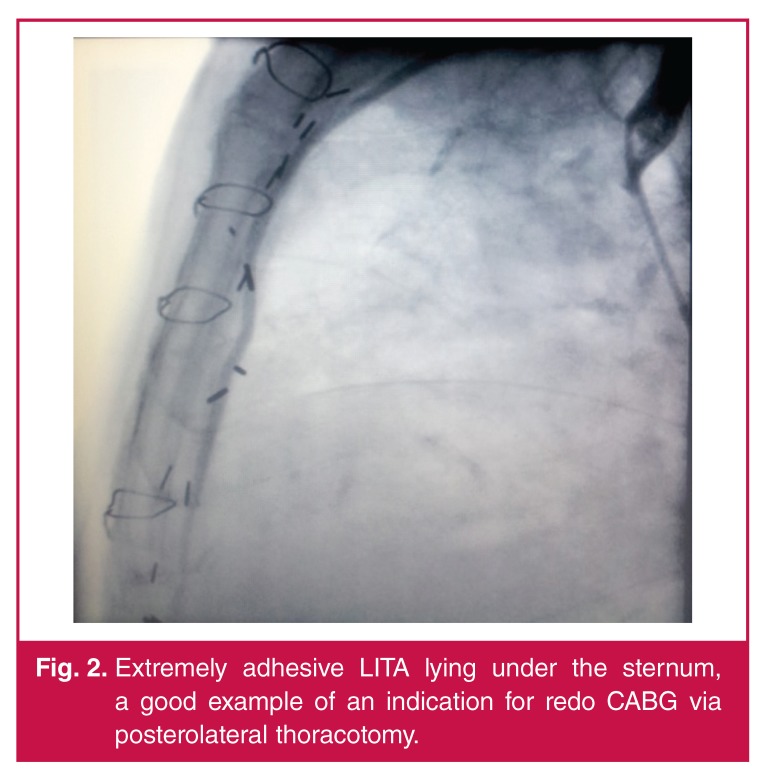
Extremely adhesive LITA lying under the sternum, a good example of an indication for redo CABG via posterolateral thoracotomy.

We decided to perform an off-pump posterolateral thoracotomy for redo CABG in these patients because of the presence of patent grafts (Fig. 2), to avoid the risks of resternotomy, and to access the posterior region of the heart more easily while revascularising the branches of the Cx system. One of the most significant independent predictors of morbidity and mortality after redo CABG is reported to be long duration of CPB.^6^ We therefore decided to avoid CPB and chose the off-pump redo CABG technique via a thoracotomy for revascularising the lateral aspect of the heart.

**Fig. 3.**
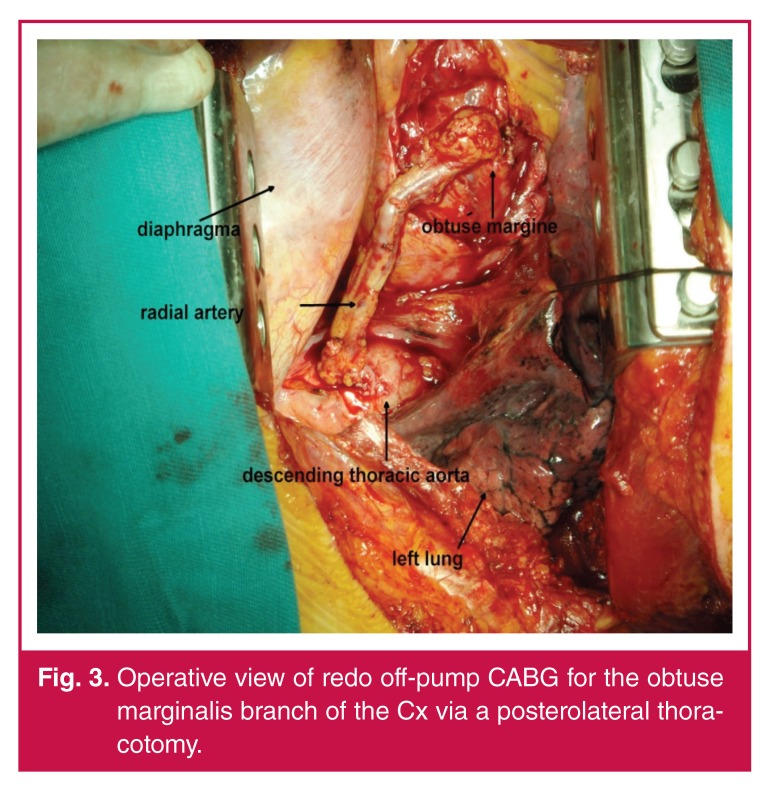
Operative view of redo off-pump CABG for the obtuse marginalis branch of the Cx via a posterolateral thoracotomy.

All patients underwent redo off-pump CABG via a left posterolateral thoracotomy (Fig. 3) with general anaesthesia after insertion of a double-lumen endotracheal tube. The patient was positioned in the right lateral decubitus position with the pelvis externally rotated slightly to allow access to the femoral vessels for cannulating the patient if necessary. The saphenous veins (SV) or radial arteries (RA) were prepared as grafts at the same time as the thoracotomy, from the contralateral extremity, without any positional problems. A supine position before thoracotomy was necessary in only one patient for harvesting the SV because the right SV had been harvested before. In six patients, the SV was harvested and in the rest the RA was used.

A left posterolateral thoracotomy was performed through the fifth intercostal space and adhesions of the collapsed left lung were dissected. After mobilisation of the left lung, the pericardium was opened above the target area, taking care with the phrenic nerve and LITA graft, which was patent in all our patients. We limited dissection of the adhesive tissues because extensive dissection may cause increased venous bleeding and a decrease in the natural stabilisation provided by the adhesions in redo CABG patients.^7^

**Fig. 4.**
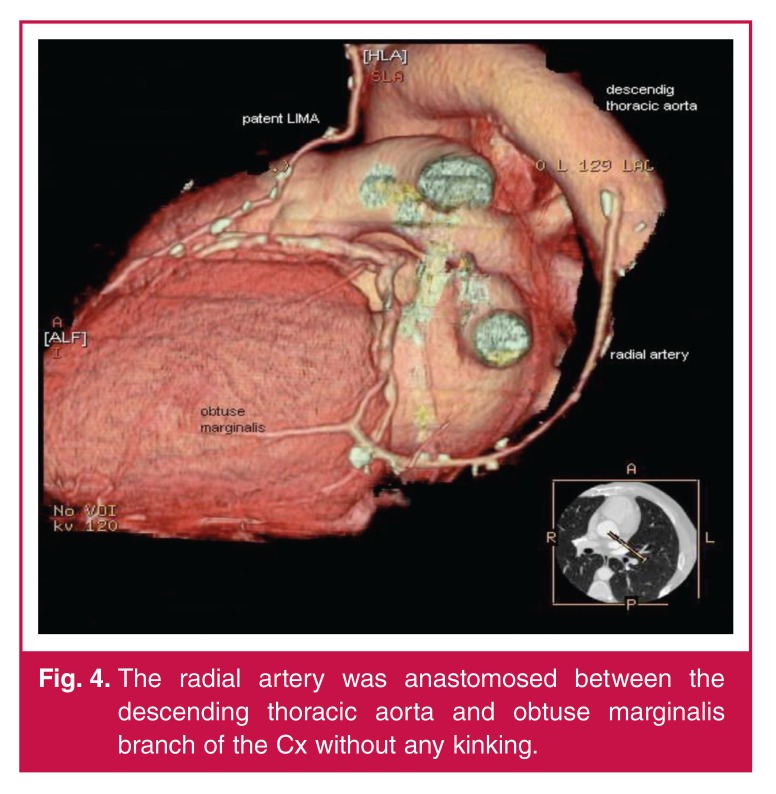
The radial artery was anastomosed between the descending thoracic aorta and obtuse marginalis branch of the Cx without any kinking.

After graft preparation, the proximal anastomosis was performed by placing a side-biting clamp on the descending aorta in a continuous fashion with a 7-0 polypropylene suture. The descending aorta was used for the inflow in all patients. When the target vessel was identified posterolaterally, four stabilising sutures were placed at each corner.^8^ After arteriotomy, to achieve a comfortable distal anastomosis, a fine vascular occlusion clamp was used to stop bleeding and the distal anastomosis was performed continuously with an 8-0 polypropylene suture. The position and length of the graft was controlled meticulously to protect it from kinking (Fig. 4).^9^

## Results

All operations were performed without CPB and electively via thoracotomy. None required conversion to resternotomy or institution of CPB. The average surgery time was 143.90 ± 36.93 minutes. The number of anastamoses was 1.1875 ± 0.39 per patient (38/32). Average ICU stay was 21.3 ± 4.41 hours with 5.08 ± 1.88 hours of respiratory assist, and drainage was 497.65 ± 291.43 ml. Average hospital stay was 5.06 ± 2.74 days (Table 3).

**Table 3:** Operative findings

*Variables*	*Mean (n = 32)*	*Min*	*Max*
Operation time (min)	143.90 ± 36.93	90	270
Drainage (ml)	497.65 ± 291.43	100	1550
Number of anastomoses	1.1875 ± 0.39	1	2
Respiratory assist (h)	5.08 ± 1.88	2	10
ICU stay (h)	21.3 ± 4.41	14	36
Hospital stay (days)	5.06 ± 2.74	4	18
Mortality	0		
Early complications			
Myocardial infarction	0		
Use of IABP	0		
(+) inotrope	1		
Atrial fibrillation	1		
Deep-vein thrombosis	1		
Thoracotomy incision infection	1		
Revision for bleeding	1		
Follow up (months)	56.17 ± 39.20	1	152
Alive and well	22		
Lost to follow up	6		
Dead	4		

The follow-up period was 56.17 ± 39.2 months (1–152) postoperatively. Twenty-two of 32 patients were alive and well, six patients were lost in the follow-up period and four patients died. There was no in-hospital mortality. All were discharged free of angina. No peri-operative myocardial infarction was observed, none of our patients required intra-aortic balloon pump (IABP) and no renal failure occurred. One patient recovered with the help of positive inotropic support. Atrial fibrillation developed in one patient, deep-vein thrombosis in another, and infection occurred in the thoracotomy incision scar of a third patient. Unfortunately one patient underwent a revision because of bleeding.

## Discussion

Redo CABG presents challenges that initial CABG surgery does not pose. Re-operative technique and the deteriorating condition of these patients cause raised morbidity and mortality rates of re-operated patients compared with the initial CABG patients.^6^ The most serious complications in isolated redo CABG are massive haemorrhage, injury to patent LITA grafts, and embolisation of the patent but very atherosclerotic ascending aorta and old venous grafts due to median resternotomy and extensive dissection of the heart.^10^-^12^

Recurrent coronary artery patients who are candidates for re-operation tend to be affected more negatively by the deleterious effects of CPB because of their decreased capacity for cardiac contractility.^6^ Off-pump redo CABG revascularising the Cx and its branches via a left posterolateral thoracotomy in carefully selected patients presents dramatically improved consequences as a result of avoiding median resternotomy and CPB.^5^,^13^,^14^

In our clinic, selected candidates for this procedure are patients suffering from angina due to lesions in the Cx and its branches, who are non-responsive to medical therapy and/or with failure of PTCA/stent. All must have patent LITA–LAD anastomoses. Other indications mentioned in the literature for this procedure are: calcified ascending but not descending aorta, sternum osteomyelitis or mediastinitis, mediastinal irradiation, requirement of concomitant left lung surgery, and previous mitral valve replacement, which creates a risk for atrioventricular groove rupture while rotating the heart to approach the arteries from the lateral aspect.^15^,^16^

We believe that re-operative off-pump CABG, performed via a left posterolateral thoracotomy to revascularise the Cx and its branches eliminates the difficulties of median resternotomy, in addition to the potential negative effects of bleeding and embolisation due to cardiac and conduit injury during extensive dissection of the heart. Avoiding resternotomy and CPB in re-operative isolated CABG surgery decreases morbidity and mortality rates.^4^,^5^

## Conclusion

In selected patients, off-pump re-operative CABG for the Cx and its branches via a left posterolateral thoracotomy can be performed with lower rates of morbidity and mortality in addition to cost-effective consequences.
